# TAARs as Novel Therapeutic Targets for the Treatment of Depression: A Narrative Review of the Interconnection with Monoamines and Adult Neurogenesis

**DOI:** 10.3390/biomedicines12061263

**Published:** 2024-06-06

**Authors:** Taisiia S. Shemiakova, Evgeniya V. Efimova, Raul R. Gainetdinov

**Affiliations:** 1 Institute of Translational Biomedicine, Saint-Petersburg State University, 199034 St. Petersburg, Russia; st035112@student.spbu.ru (T.S.S.); e.v.efimova@mail.ru (E.V.E.); 2 Saint-Petersburg University Hospital, Saint-Petersburg State University, 199034 St. Petersburg, Russia

**Keywords:** depression, antidepressant, neurogenesis, TAAR, TAAR1, monoamines, glutamate, dopamine, serotonin, SEP-363856

## Abstract

Depression is a common mental illness of great concern. Current therapy for depression is only suitable for 80% of patients and is often associated with unwanted side effects. In this regard, the search for and development of new antidepressant agents remains an urgent task. In this review, we discuss the current available evidence indicating that G protein-coupled trace amine-associated receptors (TAARs) might represent new targets for depression treatment. The most frequently studied receptor TAAR1 has already been investigated in the treatment of schizophrenia, demonstrating antidepressant and anxiolytic properties. In fact, the TAAR1 agonist Ulotaront is currently undergoing phase 2/3 clinical trials testing its safety and efficacy in the treatment of major depressive disorder and generalized anxiety disorder. Other members of the TAAR family (TAAR2, TAAR5, TAAR6, TAAR8, and TAAR9) are not only involved in the innate olfaction of volatile amines, but are also expressed in the limbic brain areas. Furthermore, animal studies have shown that TAAR2 and TAAR5 regulate emotional behaviors and thus may hold promise as potential antidepressant targets. Of particular interest is their connection with the dopamine and serotonin systems of the brain and their involvement in the regulation of adult neurogenesis, known to be affected by the antidepressant drugs currently in use. Further non-clinical and clinical studies are necessary to validate TAAR1 (and potentially other TAARs) as novel therapeutic targets for the treatment of depression.

## 1. Introduction: Pharmacotherapy of Depression

Major depressive disorder is the most common mental illness with more than 280 million cases worldwide [1,2]. The clinical picture of depression is characterized by mental disorders—anhedonia, low mood and motivation, and loss of interest and pleasure, which can lead to suicide in severe cases [3]. The disease has a huge effect on people’s standard of living, and some patients even become incapacitated and need constant care [4,5]. Apart from changes in mental state, depression in patients can be associated with cognitive impairment [6], sleep disorders [7], metabolic changes (hypercortisolemia, insulin, and leptin resistance leading to obesity, diabetes, and hypertension) [8,9]. The high prevalence of the disease, together with a negative effect on the patient’s life, makes depression a socially significant disorder, and treatment of the disease is very important in modern psychiatry.

Treatment of depression in the world of psychiatry began in the 1950s with the accidental discovery of the first substances with a positive effect on mood—iproniazid [10] and imipramine [11]. Iproniazid and imipramine are able to increase 5-hydroxytryptamine (5-HT, serotonin) and norepinephrine (NE) brain level, which is what their antidepressant effect was associated with. The increase in 5-HT and NE concentration caused by iproniazid is achieved by inhibiting monoamine oxidase (MAO), an enzyme that metabolizes these biogenic amines, and by imipramine through the non-selective reuptake inhibition of these neurotransmitters [12]. Further, other antidepressants (ADs) were discovered with similar mechanisms of action and were combined into the MAO inhibitor (MAOI) and tricyclic antidepressant (TCA) groups. MAOIs and TCAs are the first-generation ADs. Today, first-generation ADs are practically not used in psychiatry, primarily due to the severe side effects. However, their discovery served as a foundation for the monoamine theory of depression, according to which monoamine depletion leads to the development of depression [13].

Since then, other ADs have been discovered that likewise affect the activity of monoamines. They are monoamine reuptake inhibitors (MRIs) that selectively block the reverse transport of the mediator into the neuron [12]: selective 5-HT reuptake inhibitors (SSRIs), e.g., fluoxetine; selective NE reuptake inhibitors (sNRIs), e.g., maprotiline; and selective dopamine (DA) reuptake inhibitors (SDRIs), e.g., bupropion. The other agents affect the metabolism of monoamines through the inhibition of MAO-A, e.g., moclobemide. So-called atypical ADs act mainly as ligands of monoamine receptors, e.g., mirtazapine or agomelatine [14]. Also, the antagonists of presynaptic 5-HT and NE receptors (e.g., mianserin) blocking the negative feedback regulatory mechanism of synaptic levels of monoamines are used in clinics.

The monoamine theory of depression has been dominant in psychiatry for decades. Not only classical monoamines themselves, but even other metabolites of their precursor amino acids, such as kynurenines originating from tryptophan, were implicated in depression [15,16]. Monoamine ADs are widely used in clinics and for a long time remained the leaders in terms of prescription [17]. However, the experimental basis for this hypothesis remains controversial [18]. Several studies show reduced levels of monoamines and their metabolites in the blood and cerebrospinal fluid of depressed patients [19,20,21], but post-mortem brain studies of patients and healthy individuals do not always correlate with these data [22]. Currently, a reliably confirmed point of the monoamine hypothesis is that the lack of 5-HT, DA, and NE does indeed worsen the course of the disease in depressed patients or those in remission, but is not capable of leading to depression, especially without burdened heredity [23]. In 2022, a comprehensive umbrella review was published refuting the link between 5-HT and depression and demonstrating no support for the hypothesis that depression is caused by lowered 5-HT activity or concentrations [24].

In addition, unresolved issues remain in the work of monoamine ADs. First of all, there is a clinical effect in the several weeks following the onset of drug taking. Second, a lot of patients (20–30%) with depression are resistant to treatment with these medications [15]. Moreover, even in the case of successful therapy, there is no guarantee that the patient will not develop resistance following long-term treatment [25].

These problems were solved by introducing ADs with a non-monoamine mechanism of action into clinical practice. In the 1990s, it became known that antagonists of the N-methyl-D-aspartate (NMDA) receptor, an ionotropic glutamate receptor, exhibit antidepressant activity [26]. Later, ketamine and esketamine, as well as other NMDA receptor antagonists, demonstrated rapid and long-lasting antidepressant effects for treatment-resistant depression [27].

In 2019 the first-in-class rapid-acting AD esketamine was approved by the Food and Drug Administration (FDA). The esketamine mechanism of action as well as ketamine (used off-label as AD) and dextromethorphan in combination with bupropion, recently approved by the FDA under the brand name Auvelity, seems to be associated with the blockade of NMDA receptors. These ADs are effective in the case of many patients resistant to the ”traditional” ADs. These ADs are characterized by a fast onset of action and can be used in patients with a high risk of suicide. Despite the success of NMDA antagonists, it is important to understand that these agents can induce serious adverse reactions including addiction [28] and toxicity [29].

There is no universal treatment for depression, nor a mechanism that explains all aspects today. Due to this, the search for new drug targets and the study of pathogenesis still remains a relevant task. Currently, several substances are being tested in clinical trials for depression treatment. These include both agents affecting monoamine neurotransmission and those involving other systems [30]. As can be seen, pharmacotherapy for depression is gradually moving beyond the monoamine hypothesis, and unresolved difficulties stimulate the creation of new drugs.

In this review, we propose to consider trace amine-associated receptors (TAARs) as new promising pharmacological targets for the treatment of depression. Today, the study of TAARs is at the peak of its popularity, so it is very important to systematize existing information and provide new hypotheses. While there are excellent reviews showing the great potential of TAAR1 agonists in the treatment of depression [31,32], we extend these observations by including the most recent data on TAAR1 agonists and showing the potential of other TAARs as novel targets for the treatment of depression.

Here, we focused on the relationship of TAARs not only to monoamines, but also to adult neurogenesis, showing that all three systems mutually influence each other and contribute to the development and/or treatment of depression. Thus, we propose to direct further research in this area, considering it very promising. In addition, we go beyond the usual study of TAAR1 and show the potential of other receptors.

## 2. Neurogenesis and Its Association with Depression

**Figure 1:**
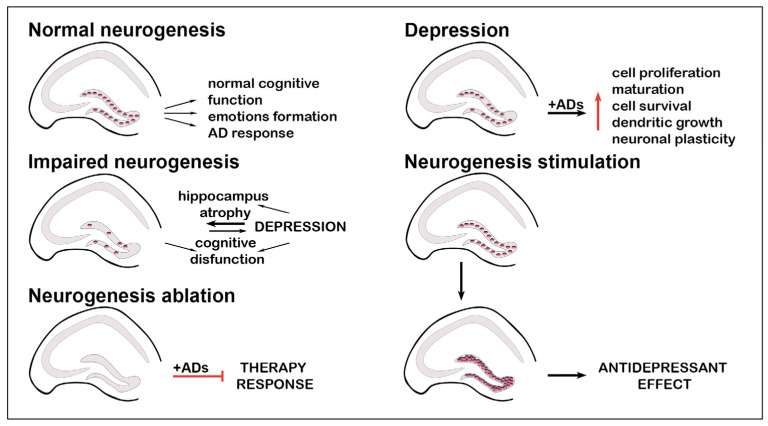
Interconnection between neurogenic processes and depression. Adult hippocampal neurogenesis is associated with emotion formation and cognitive function. Moreover, healthy adult neurogenesis is essential for response to antidepressant treatment. Impaired adult neurogenesis is associated with depression and cognitive decline. The priority of the processes is not yet clear. In the complete absence of adult neurogenesis, treatment with antidepressants is not effective. Antidepressant treatment enhances adult neurogenesis by increasing cell proliferation, maturation, and survival. The stimulation of adult neurogenesis leads to an antidepressant effect.

Some gaps in the existing depression hypothesis can be filled by a theory linking altered hippocampal neurogenesis with the development of depression (Figure 1). Increasingly, the hippocampus is considered an area involved in the pathogenesis of depression and associated with cognitive and emotion formation processes. Moreover, healthy neurogenesis is required for depression treatment [33]. A lack of response to both drug therapy for depression (fluoxetine and imipramine) and alternative therapy (electroconvulsive antidepressant therapy [34], intermittent hypobaric hypoxia [35]) during neurogenesis ablation in animal models has been shown [36,37]. AD treatment (5-HT and NE reverse inhibitors) promotes neurogenesis by increasing cell proliferation, maturation, cell survival, dendritic growth, and neuronal plasticity [38]. In addition, neurogenesis can directly exert an antidepressant effect. The protective potential of activating and maintaining neurogenesis agents has been shown in mouse models of depression. For example, baicalin, a flavonoid with anti-inflammatory, anti-apoptotic, and neuroprotective functions, has demonstrated an antidepressant effect in the chronic unpredictable mild stress model [39].

The link between hippocampal neurogenesis and depression can be inferred in humans as well. It is known that cognitive dysfunction, in particular memory impairment, is a clear sign of depression. Studies of patients suffering from depression have revealed a decrease in the volume of the hippocampus, correlated with the severity and duration of the disease [40,41,42]. With stress-induced atrophy of the hippocampus, a decrease in the number of cells can contribute to the development of depression.

Having discussed the main points of the connection between depression and neurogenesis, it is important to note the role of monoamine systems in both of these processes. Monoamines are able to up- or downregulate neurogenesis by activating the corresponding G protein-coupled receptor (GPCR). As a rule, a decrease in cyclic adenosine monophosphate (cAMP) level through the activation of the Gi protein or an increase of phospholipase C through the activation of Gq leads to proliferative processes, while the stimulation of receptors associated with the activation of the Gs protein directs cells to the path of differentiation [43]. More recent studies show that the regulation of neurogenesis is related to the balance, or the ratio of up- and downregulating receptors involved in neurogenesis [44,45]. Receptors that stimulate different stages of neurogenesis include NE receptors (α1 [46], β3 [47]), DA receptors (D2-like receptors) [48], and 5-HT receptors (5-HT1A) [49].

Neurogenic responses to ADs are also associated with the activation of certain monoamine GPCRs. Fluoxetine upregulates 5-HT1A receptors together with neurogenesis, suggesting that these processes could be related [33]. Rivastigmine activates neurogenesis and alleviates symptoms of depression in a mouse bulbectomy model by engaging the serotonin 5-HT1A receptor [50]. The β3-adrenergic receptor promotes the activation of neurogenic progenitors and stem cells [47]. During the activation of the D1 receptor by agonists, a neurogenic effect was observed, namely the increased proliferation and the survival of progenitor cells in the hippocampus of adult rats [51].

It is important to note that it is a chronic, but not acute, antidepressant treatment that has a neurogenic effect, which is consistent with the dynamics of human recovery. Moreover, the progress of the neurogenic process correlates with the success of therapy (rats). It has been shown that it is chronic rather than acute and subchronic, fluoxetine treatment which produces both antidepressant and neurogenic effects [52].

Together, the described data indicate the relationship between the development of depression and a decrease in neurogenesis. However, information is not yet sufficient to establish if neurogenesis is the cause of the development of depression or a consequence or just a coincidence. The neurogenic theory of depression may fill in the gaps in the monoamine theory. For example, to explain the delayed effect of ADs, it takes time to turn on neurogenic processes. It is possible that new hippocampal cells are able to overcome depression-induced atrophy [53] and serve as a new resource for the activation of brain plasticity and relearning, thus being the missing link in the response to ADs.

As depression is a complicated and heterogeneous disease with a complex etiology, more than one system is likely involved in its development. With further studies, more information is gathered suggesting that the monoamines and neurogenesis are not the only systems that are altered in depression and there could be other brain processes involved, possibly by influencing both those systems.

## 3. The Family of TAARs—A New Target for Depression Therapy?

### 3.1. Trace Amines

One of the intriguing players potentially involved in mood regulation is an endogenous compound’s group of trace amines (TAs). TAs such as beta-phenylethylamine, tyramine, tryptamine, octopamine, synephrine, and many other biogenic amines are present in mammalian tissues at nanomolar (0.1–10 nM) trace concentrations [54,55]. Generally, many TAs are the products of decarboxylation of precursor amino acids by the enzyme aromatic L-amino acid decarboxylase and are metabolized by MAO-A and MAO-B [56]. In addition to being structurally similar to classical biogenic amines (DA, 5-HT, and NE), TAs are metabolically closely related to these neurotransmitter systems, where they are widely present. Along with these mediators, TAs seem to participate in the regulation of emotional behaviors, mood, thoughts, or perception [57,58,59].

TAs can function as neurotransmitters within their own unique signal transduction system, but in the aspect of depression, the work of TAs as co-transmitters and modulators of classical monoamines is intriguing [52]. At their physiological concentration, TAs are able to change a cell’s responses to other neurotransmitters [56]. The modulation of postsynaptic transmission of NE and DA by beta-phenylethylamine and the potentiation of NE and DA responses in neurons by tryptamine are already well known [59,60]. Due to the ability of TAs to affect monoamine neurotransmission and their presence in monoamine regions of the mammalian brain, they are of great interest to psychiatry.

To clarify the role of TAs in the pathogenesis of depression, a number of studies were conducted to study the content of TAs and their metabolites. Thus, several studies have demonstrated that in depressed patients suffering from bipolar affective disorder, urinary excretion of beta-phenylethylamine is reduced [61,62,63,64], while in patients in the manic phase, on the contrary, it is increased [65]. Phenylethylamine deficiency in people with depression was confirmed by examining their cerebrospinal fluid. A reduced content of phenylacetic acid, a metabolite of beta-phenylethylamine, was found in the cerebrospinal fluid [66]. A similar decrease in the content of some other TAs in the blood plasma, cerebrospinal fluid, and urine of patients with depression is also known [67,68,69]. In turn, treatment with TCAs (clomipramine) in depressed patients who had reduced renal excretion of phenylethylamine led to both the disappearance of clinical symptoms and an increase in beta-phenylethylamine levels to normal values [62,65]. Furthermore, the use of beta-phenylethylamine precursor phenylalanine alone or in combination with other ADs led to progress in therapy in previously unresponsive patients [70]. It is worth noting that there are a sufficient number of studies in which researchers were unable to detect a connection between affective diseases and the TA content in biological fluids [71]. For example, renal excretion of tryptamine increased during treatment with imipramine in patients with depression, but it was not possible to identify a correlation between an increase in the level of this amine and an improvement in the clinical picture [72]. Based on these data, one can speculate about the role of TAs in the pathogenesis of depression. These data even gave rise to a hypothesis about the involvement of beta-phenylethylamine, and later other TAs, in the formation of depression [71]. However, these research projects were carried out in the second half of last century, and the results are multidirectional and contradictory, so a comprehensive, uniform, and detailed study of this issue is required.

### 3.2. TAAR1 Agonists Are New Generation Antipsychotics

The study of TAs reached a new level after the discovery of the so-called trace amine-associated receptors, TAARs. TAARs are a family of GPCR receptors that induce the classical cAMP cascade and activation of downstream targets. In vertebrates, there are nine TAAR subfamilies expressed both in the central nervous system and in the periphery [49]. TAARs have functional interspecies differences due to pseudogenization events and species-specific expansions. In humans, there are six functional types of TAARs—TAAR1, TAAR2, TAAR5, TAAR6, TAAR8, and TAAR9 receptors, with TAAR3, TAAR4, and TAAR7 receptors being pseudogenes [73]. The genes encoding TAARs form a cluster on chromosome 6 at band q23.2, which has been identified as a susceptibility locus for schizophrenia in humans [74].

The best known and most frequently studied of those, TAAR1, is already being actively investigated in the aspect of mental and neuropsychiatric disorders [75]. The ability of TAAR1 to regulate DA and 5-HT as well as glutamatergic neurotransmission formed the basis of this interest [55]. Moreover, TAAR1 is widely represented in the limbic and monoamine systems of the brain, which are responsible for psychotic states, mood, attention, memory, fear, and addiction [73]. In addition, *taar1* mutations disrupting the receptor’s function were found in people diagnosed with schizophrenia [76]. It is possible that carriers of such genes are more at risk of schizophrenia and require activation of the subfunctional receptor [77].

The discovery of selective TAAR1 agonists made it possible to elucidate the functional significance and therapeutic potential of the receptor in more detail. Starting with RO5256390, new partial or full TAAR1 agonists with high affinity and selectivity for TAAR1 have been created and studied [78]. Initially, TAAR1 agonists were considered for the treatment of schizophrenia [79,80]. Researchers have already shown their effectiveness against the positive, negative (lack of motivation, anhedonia), and cognitive symptoms of schizophrenia, which are often overlooked by typical antipsychotics [64]. The most successful new psychotropic drug is SEP-363856 (SEP-856, trade name Ulotaront), a TAAR1 receptor agonist with low 5-HT1A activity [81]. Ulotaront has already passed the second phase of clinical trials in the treatment of schizophrenia, which led to FDA designation as a Breakthrough Therapy for this indication [82]. The unique mechanism of action avoids the side effects of typical D2 antagonist antipsychotics (extrapyramidal symptoms, weight gain), reduces substance abuse cravings, and relieves depressive symptoms [83]. Thus, therapy based on TAAR1 activation has proven to be an excellent alternative to antipsychotics for patients who do not respond to therapy or refuse it due to the severe side effects. Early evidence suggests that TAAR1 activation does not cause the side effects associated with typical antipsychotics [84]. However, more studies are needed to ascertain their safety and tolerability.

### 3.3. TAAR1 Agonists Are New Generation Antidepressants

Today, TAAR1 agonists have already been comprehensively studied and are of great value in the aspect of drug addiction, mental and metabolic diseases. Interestingly, it was found that TAAR1 agonists also exhibit useful properties for the treatment of depression [31]. For instance, TAAR1 agonists RO5256390, RO5203648, and RO5263397, in addition to antipsychotic actions, have demonstrated in vivo improvement in the sleep–wake cycle [85,86], reduction in drug cravings [87,88], and procognitive properties [89,90,91]. The direct antidepressant potential is indicated by work on the forced swimming test in rodents. It was shown that RO5263397 and RO5203648 treatment led to dose-dependent immobility time reduction in forced swimming tests [86,87,88]. The TAAR1 full agonist RO5256390 was not found to have any effects on depressive-like behavior in the same test [92].

It is significant that Ulotaront has also demonstrated an antidepressant effect in rodent and non-human primate tests [93]. In rats, behavioral tests of Ulotaront have shown that its efficacy in attenuated social withdrawal is comparable to that of clozapine. In forced swimming tests, mice have demonstrated immobility time reduction. On par with the above-mentioned agonists, Ulotaront exerts REM sleep suppression, improving the sleep–wake cycle [94]. Ulotaront is currently undergoing a phase 2/3 clinical trial testing its safety and efficacy in the treatment of major depressive disorder and generalized anxiety disorder and in adults [95]. In addition to the good isolated effect of Ulotaront, in combination with Duloxetine (5-HT and NE dual reuptake inhibitor), tests showed better results in experimental animals. Therefore, the synergy of TAAR1 and monoamines can be a powerful tool to improve AD action [96].

Another TAAR1 agonist, o-PIT (o-phenyl-iodotyramine), also confirms the antidepressant potential of TAAR1. In forced swimming tests, wild-type mice (but not TAAR1 knockout (KO) mice) have demonstrated immobility time reduction in a dose-dependent manner [97].

Based on data from the acute and chronic administration of RO5256390, the TAAR1 activation antidepressant effect is supposed to be associated with increased 5-HT and DA neurotransmission in the dorsal raphe nucleus and the ventral tegmental area, respectively. During acute exposure of RO5256390, an increased extracellular 5-HT and DA leads to activation of the 5-HT1A and D2 receptors, and during chronic exposure, desensitization of these receptors occurs [98]. Interestingly, the same mechanism of action is characteristic of monoamine ADs. For example, the acute administration of SSRIs also inhibits, and chronic administration stimulates, the firing rate of 5-HT neurons [99].

In addition, convincing evidence is emerging that TAAR1 is involved in the regulation of neurogenesis. According to transcriptomic data, TAAR1 is expressed in the murine and human hippocampus [100]. The ligands of TAAR1, beta-phenylethylamine and T1AM (3-Iodothyronamine), were shown to have a positive effect on neurogenesis [101,102]. Phenylethylamine is able to regulate BDNF levels and restore the number of hippocampal dendritic spines in the cortisol-induced depression mouse model [103].

Another piece of evidence comes from recent work showing a connection between TAAR1, neurogenesis, and depression [104]. The study demonstrated that TAAR1 in the hippocampal dentate gyrus mediates the effects of chronic stress on neurogenesis, hippocampal plasticity, and cognitive function in mice. Mice in the chronic social defeat stress model had reduced levels of TAAR1 mRNA and impairments in hippocampal neurogenesis and cognitive function. Interestingly, the effects of stress were neutralized by the administration of the TAAR1 agonist, RO5263397. Moreover, selective knockout of the taar1 gene in the dentate gyrus mimicked cognitive and neurogenic deficits caused by chronic stress [104].

In summary, although TAAR1 agonists are primarily considered antipsychotics, they have significant antidepressant potential. As the direct antidepressant effect has been shown in a few tests, they have demonstrated a line of useful properties for depression treatment. Among them are procognitive functions and improved sleep. Moreover, TAAR1 agonists attract the attention of clinicians due to the absence of severe side effects. Therefore, it is advisable to continue studying the antidepressant properties of TAAR1 and other TAARs.

### 3.4.“Olfactory” TAARs in the Treatment of Depression: Perspectives

**Figure 2:**
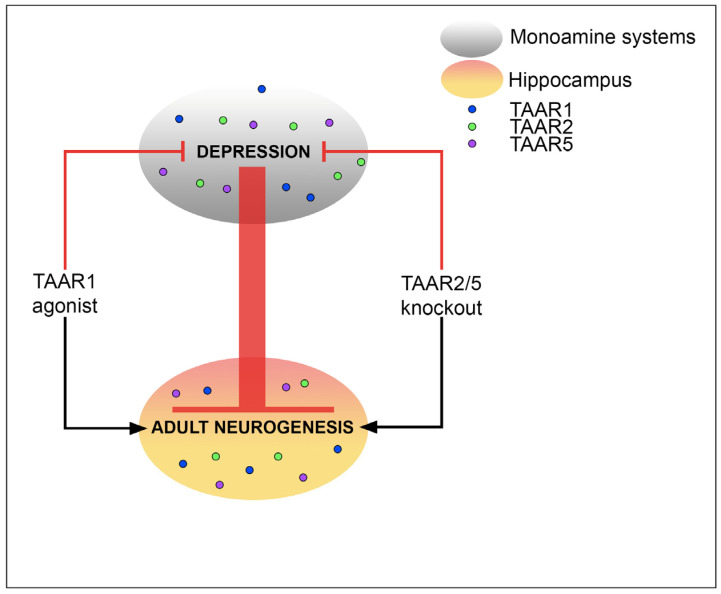
TAARs in depression and adult hippocampal neurogenesis. The pathogenesis of depression is closely related to the monoamine systems of the brain. TAAR1, TAAR2, and TAAR5 were found in the monoamine nuclei of the brain and in the hippocampus, the center of neurogenesis. Depression inhibits adult neurogenesis. TAAR1 agonists have an antidepressant effect and promote neurogenesis. TAAR2 and TAAR5 knockout animals exhibit decreased depressive-like behavior and increased adult neurogenesis.

The remaining TAARs (TAAR2-TAAR9) are primarily known as olfactory receptors sensing innate odors mediated by volatile amines originating from the decarboxylation of amino acids [105]. They are found in the olfactory epithelium and olfactory bulbs of mammals and activate innate behaviors. However, as studies progress, it becomes clear that the effect of “olfactory” TAARs is not limited to the detection of volatile and aversive amines from outside of the body and, along with TAAR1, can become a target for the treatment of mental illness, in particular depression (Figure 2).

Gradually accumulating transcriptomic data makes it possible to associate TAARs with the regulation of emotions, as they were shown to be expressed in the limbic areas [100]. Also, based on transcriptome data, it is hypothesized that TAARs may be involved in the pathogenesis of mental illness [100,106]. Several studies have identified the association of not only *taar1* but also *taar2*, *taar5*, and *taar6* SNPs (single nucleotide polymorphisms) with schizophrenia and bipolar disorder [77]. Mutations in the *taar6* gene may be associated with the severity of depression and the effectiveness of response to therapy [107]. Moreover, it turned out that TAAR5 expression in the prefrontal cortex may be impaired in patients with depression [108]. Thus, “olfactory” TAARs are of great interest for a more detailed and large-scale study in the context of mental illness.

TAAR2 and TAAR5 are currently the most studied of the “olfactory” TAARs and appear to have similar functional significance. To date, TAAR2 and TAAR5 have been found not only in the olfactory system but also in the limbic region of the mammalian brain and some monoamine nuclei. Histochemical methods have shown that both TAAR2 and TAAR5 are expressed in the hippocampus, the nuclei of the thalamus and hypothalamus, and the piriform cortex. In addition, TAAR5 was found in the amygdala, orbitofrontal cortex, nucleus accumbens, entorhinal cortex [109], and neurogenic niches—the subventricular zone [110]. TAAR2 was found in the lateral habenula and raphe nuclei [111]. The expression of other TAARs (most prominently TAAR5 and TAAR6) in the murine and human hippocampus and other limbic regions was also documented based on the analysis of public transcriptomic data [100,108,112]. In further studies in knockout (KO) mice, other properties of TAAR2 and TAAR5 were found that bring them closer to the pathogenesis of depression. There is accumulating evidence of a relationship between the TAARs and the monoamine system. Of particular interest is the increased DA level and its metabolites in the striatum in TAAR2-KO and TAAR5-KO mice, as well as the increased number of tyrosine hydroxylase-positive neurons in the substantia nigra pars compacta. These changes complement the increase in the content of growth factors: BDNF in the striatum in TAAR2-KO and GDNF in TAAR5-KO [110,111]. Behavioral changes in TAAR KO mice also support their emotional role and association with monoamines. In particular, TAAR5-KO and TAAR2-KO mice exhibited less anxious and depressive behavior in several behavioral tests [109,110]. Behavioral changes might also be connected to the 5-HT system. In TAAR5-KO mice, the level of 5-HT in the striatum and hippocampus is reduced, and 5-hydroxyindoleacetic acid is reduced in the hippocampus and hypothalamus. It is thus possible that the lack of TAAR5 affects the functional state of the brain 5-HT system [109]. This relationship is confirmed by the altered cognitive profile of TAAR5-KO. TAAR5-KO mice showed fewer errors, better execution speed, and higher learning progress [113].

Perhaps the most intriguing characteristic of TAAR2-KO and TAAR5-KO mice is the increased adult neurogenesis. In both models, an increased number of neurogenic markers, namely proliferating cell nuclear antigen (PCNA), and doublecortin (DCX) in the subventricular and the subgranular zone, were found compared to wild-type [107,108]. Intriguingly, the expression of several TAARs including TAAR2 and TAAR5 was found during the differentiation of human pluripotent stem cells to dopaminergic neurons [114].

The discovery of TAAR expression in the limbic and monoamine regions opens up new possibilities for their use for therapeutic purposes. However, one should not forget about their predominantly olfactory localization. It is the simultaneous representation of TAARs in both the olfactory and limbic systems that may be a key advantage for the treatment of mental illness, primarily depression. The olfactory system plays an important role in the functioning of the limbic system. By receiving olfactory inputs, the limbic region of the brain corrects emotional responses and species-specific behavior [115,116]. The relationship of these two systems is also indicated by the consequences of bulbectomy in mice. Removal of the olfactory bulbs leads to the manifestation of depression-like behavior and a decrease in neurogenesis [117]. At the same time, anosmia in humans is an early prognostic sign of various neurodegenerative and mental diseases and their symptoms [118]. Interestingly, TAAR2- and TAAR5-KO mice exhibit anti-anxiety and antidepressant behavior concomitantly with increased neurogenesis. The only non-selective TAAR5 agonist known to date, α-NETA, induces psychotic-like episodes in mice [119]. Such consequences of the absence of TAARs suggest the antidepressant potential of their antagonists. The search for such TAAR5 antagonists has already started [120].

Together, these data can be justification for considering “olfactory” TAARs and related neurotransmitter systems a new target for depression therapy and the further study of them in terms of this disease [121].

## 4. Conclusions

The accumulated data allow us to consider TAARs a promising new target for the treatment of mental and nervous diseases. Agonists of TAAR1, the most frequently studied of the receptors, are already in clinical trials as an antipsychotic, antidepressant, and anxiolytic drug. Exposure to TAAR1 agonists causes an antipsychotic effect but is not accompanied by serious side effects characteristic of typical antipsychotics. In addition, they have shown high efficacy against the negative and cognitive symptoms of schizophrenia, including the antidepressant effect. Other members of the TAAR family, the so-called “olfactory” TAARs, have also shown antidepressant potential. Animal studies have shown that TAAR2-KO and TAAR5-KO have an anti-anxiety and antidepressant phenotype and, like TAAR1, have a modulating effect on the brain’s monoamine systems. In addition, the localization of TAARs in the limbic and monoamine systems of the mammalian brain, which are associated with the formation of emotions and mood, motivation, and cognitive functions, has recently become known. Against the background of these facts, changes in neurogenic processes during the blockade of TAAR1, TAAR2, and TAAR5 seem to be an extremely intriguing and promising detail. In summary, a detailed study of TAARs could help clarify aspects of depression pathogenesis and identify cause-and-effect relationships in its development. Probably, TAARs are in fact the missing link between depression, neurogenesis, and the monoamine system.

In addition, drugs based on TAAR agonists could become a panacea for vulnerable groups of the population, adolescents, the elderly, and pregnant women. For these groups, mental disease therapy is associated with risks and remains poorly studied. Given the good tolerability of TAAR1 agonists, it is necessary to expand the study of their use. The absence of serious side effects makes these drugs extremely attractive. Thus, it is necessary to continue the study of TAARs, and to look for TAAR agonists and antagonists to better understand their therapeutic properties.

## Data Availability

Not applicable.
